# Hepatitis C Avoidance in Injection Drug Users: A Typology of Possible Protective Practices

**DOI:** 10.1371/journal.pone.0077038

**Published:** 2013-10-23

**Authors:** Catherine McGowan, Magdalena Harris, Tim Rhodes

**Affiliations:** Department of Social and Environmental Health Research, London School of Hygiene & Tropical Medicine, London, United Kingdom; University of Stirling, United Kingdom

## Abstract

**Introduction:**

Hepatitis C virus (HCV) represents a serious public health concern. People who inject drugs (PWID) are at particular risk and nearly half (45%) of PWID in England may be infected. HCV prevention interventions have only had moderate impact on the prevalence of HCV in this population. Using qualitative methods, we sought to detail the protective practices potentially linked to HCV avoidance among PWID, and explore the motivations for these.

**Methods:**

The study used a life history approach allowing participants to detail their lived experience both before and during the course of their injecting careers. Thirty-seven participants were recruited from drug services in London, and from referrals within local injecting networks. A baseline and follow-up in-depth qualitative interview was carried out with each participant, and for half, a third interview was also undertaken. All underwent testing for HCV antibody. Analyses focused on developing a descriptive typology of protective practices potentially linked to HCV avoidance.

**Results:**

Practices were deemed to be protective against HCV if they could be expected *a priori* to reduce the number of overall injections and/or the number of injections using shared injecting equipment. Participants reported engaging in various protective practices which fell into three categories identified through thematic analysis: principles about injecting, preparedness, and flexibility.

**Conclusions:**

All participants engaged in protective practices irrespective of serostatus. It is important to consider the relative importance of different motivations framing protective practices in order to formulate harm reduction interventions which appeal to the situated concerns of PWID, especially given that these protective practices may also help protect against HIV and other blood borne infections.

## Introduction

Approximately 216, 000 individuals in the UK are living with chronic hepatitis C virus (HCV). [1] Regional estimates suggest that, in England, 45% of people who inject drugs (PWID) are living with chronic HCV. [1] The prevalence estimates for PWID in Wales (39%), Northern Ireland (29%), and Scotland (55%) are also high. [1] HCV-related admissions to hospital have risen threefold (from 612 in 1998 to 1, 979 in 2010), as have HCV-related deaths (from 98 in 1996 to 323 in 2010). [1] In London the prevalence of HCV among PWID is 56% (CI 51%–62%) and among former PWID, 39% (CI 33%–46%). [1].

The strongest predictor of HCV infection is a history of injection drug use. [1], [2] A recent study has suggested that 85% of those with chronic HCV infection are either current or former PWID. [2] Specific risk factors for HCV exposure among PWID include: sharing needles, sharing other injection equipment (*e.g.* cookers and filters [3], [4]), frequency of injection [5], front loading [6], being injected by another [7], injection cocaine use [8], lack of treatment for dependence [5], [9], being female (though the prevalence of HCV in England and Wales is higher in males [2]) [7], and the length of time since first injection. [5], [8], [10] Structural factors, such as incarceration [9], [11] and homelessness [12], have also been correlated with HCV seropositivity.

Annual reports produced by the UK Health Protection Agency emphasise the prevention of new infections as a priority. [1], [13] Opiate substitution therapy and needle exchange programmes are the most common primary prevention methods aimed at reducing transmission of HCV. Though needle exchange programmes (NEP) have been shown to reduce the incidence of HCV, opioid substitution treatment (OST) has only demonstrated marginal effectiveness.[14]–[16] However, evidence from the Amsterdam Cohort Studies suggests that full participation in harm reduction programmes (defined as participating in NEP and OST concurrently) is associated with a decrease in incident HCV infections when compared to no participation (incidence rate ratio 0.43 [95% CI 0.21–0.97]). [17].

Other prevention strategies include behavioural programmes and syringe bleaching, neither of which have produced convincing results. [14] A 2009 study assessing the efficacy of a series of motivational interventions aimed specifically at raising awareness of behaviours which put PWID at risk of HCV (with the explicit goal of avoiding HCV seroconversion) showed no effect when compared to the control population. [18] Thus, with the exception of needle exchange programmes, and the marginal efficacy of OST, the remaining policy interventions have been ineffectual in reducing exposure, and subsequent seroconversion, to HCV. [14] Needle exchange programmes, whilst effective in reducing incident infections, have had little impact on the overall prevalence. [14] This is despite these harm reduction strategies being proven effective for reducing HIV infection. [19].

Several qualitative studies have suggested that the inefficacy of the interventions to prevent HCV rests not on their inability to reach the target population, or their inability to offer sound strategies to avoid infection, but rather on failure to recognise the weak motivational force of HCV avoidance.[20]–[22] Despite the fact that current guidance for drug treatment providers in the UK emphasises strategies to help PWID avoid HCV infection [23], this approach is unlikely to result in a significant decrease in incident infections; therefore, it is clearly of interest to policy makers to discover those practices that are likely to facilitate HCV avoidance, what motivations are responsible for such practices, and what circumstances frustrate these practices.

We report on a qualitative study which sought to understand the drug use and social practices associated with long-term viral avoidance, and to consider how such practices are shaped by the life trajectories of PWID, and their social contexts over time. The study was innovative through its use of life history methods to understand and detail the lived experience of people who have been injecting long-term. It offers to inform our understanding of practices that have protective potential with respect to HCV. What follows is a typology of protective practices identified across the interview accounts of the 37 PWID who participated in the study. We also explore the motivations and interpretative frameworks shaping these practices, and how they may offer a putative protective advantage against HCV infection.

## Methods

### Participants

Participants were referred from collaborating HCV testing and screening services in South East and North London. At each recruitment location a Primary Care Physician contacted eligible participants to pass on both the participant information sheet and the contact details of a member of the research team. Participants were also recruited through referral within drug user networks. Purposive sampling was employed in order to elicit the participation of both male and female PWID who experienced differing: durations of injection drug use, housing situations, injection drug preferences (*i.e.* heroin, crack cocaine, and ‘speedballs’), and (in the case of HCV positive participants) the length of time since HCV diagnosis. This sampling strategy resulted in the inclusion of participants of different ages, ethnic backgrounds, and relationship status. Those included in the sample also had varying experiences regarding: incarceration, preferred injection location (*e.g.* groin), therapeutic methadone use, and inpatient drug treatment.

The sample included 37 individuals, all of whom were invited for a second interview. A third interview was requested of half those attending the second interview. All participants were articulate and able to describe in great detail their injection practices, their motivations for engaging in such practices, and the conditions under which these practices are frustrated.

### Data Collection

Data were collected between January 2010 and August 2011 by the principal investigators (MH and TR). All participants consented to an initial in-depth interview lasting approximately two hours. This baseline interview was designed around a life history approach and entailed the creation of life-grids to map participants’ life trajectories. [24] The creation of the life-grids was participant-led. Most participants focused on significant life events, and ongoing experiences with injection drug use. Timeline Maker™ software was used to create visual representations of the life-grids and to facilitate case comparisons revealing potential relationships between life events and patterns of injection practices, risk avoidance, and those factors which facilitate risk.

Following the initial interview participants were invited to submit to serological screening for HCV. Once they had completed HCV testing, participants were invited for a second interview. Interviewers were informed of the test results prior to the second interview. These follow-up interviews pursued key narrative themes identified in the first interview while focusing across cases to explore: the development of risk aversion, the perceived constraints to risk avoidance presented by particular conditions or situations, and the properties and dimensions of prophylactic drug use practices in context and over time. A third interview was conducted as a means to further pursue participant narratives deemed to be particularly information-rich.

### Data Analysis

All interviews were audio recorded (with participant consent) and transcribed. Drawing across life history and qualitative interview data, both within and across cases, our analysis reported here focused primarily on systematically identifying a descriptive typology of protective practices potentially linked to the avoidance of HCV in the long-term. Interviews were coded as they were collected in order to inform the direction of subsequent interviews. Coding was carried out by two members of the research team. Codes were compared between researchers and points of divergence were discussed at length in order to maximise internal reliability. Drawing upon inductive and grounded analysis techniques, we also coded accounts for key themes linked to practices potentially HCV-protective in order to explore participant interpretative frameworks and the motivations shaping these. Protective practices fell broadly into three categories: *principles*, *preparedness,* and *flexibility* concerning drug use.

### Ethics Statement

Ethical approval for the study was obtained from the Research Ethics Committee at the London School of Hygiene & Tropical Medicine and the North London Regional Ethics Committee. The recruitment materials explicitly indicated that participation in the study was voluntary, anonymous, and that the results of the serological screening would be confidential. A separate consent form was issued to participants who agreed to undergo serological screening for HCV antibodies, and HCV RNA. All participants provided written consent to be interviewed, and to participate in HCV screening. Counselling services were provided at the participating HCV testing centres to inform participants of the results and to answer questions.

## Results

Twenty-seven of the participants (73%) were men and 10 (27%) were women. All of the participants were long-term users (defined as having injected for six years or longer). The average age of participants was 41 years (23–57 years). Twenty-five participants (68%) had spent time in prison, 20 (54%) had been previously admitted to a rehabilitation or detoxification facility, 35 (95%) were either receiving OST at the time of the interview or had at some point in the past, and 33 (89%) were either on a break from injecting or had taken breaks from injecting in the past.

All 37 participants underwent testing for HCV, with 22 (59%) testing seronegative, and 15 (41%) seropositive. Many of those who were found to be HCV-positive were consistently and conscientiously engaging in practices deemed theoretically protective. Practices were deemed protective if they could be expected to reduce the total number of injections (*e.g.* when reverting to smoking/snorting rather than injecting), or when the practice limited or eliminated injections using shared equipment (*e.g.* separating or marking of equipment or having rules about not sharing needles/syringes). Participants often reported engaging in several different protective practices.

### A Typology of HCV Protective Practices

The protective practices identified across participant interview accounts fell broadly into three distinct, but related, thematic categories: *principles*, *preparedness*, and *flexibility* concerning the enactment of drug use practices. We defined the category ‘*principles*’ to include those normative practices which were routinely and often rigidly adhered to by participants. Principles were most often described prescriptively (*e.g.* “I actually had rules, it was [to] use clean needles…clear up after yourself…always” [C05]); though proscriptive principles were not uncommon (*e.g.* “I’m not sharing needles! - blatant, I’m not sharing. If it hurts your feelings, well tough, I’m not sharing” [C04]). Principles did not have to be inspired by specific motivations and could be described axiomatically (*e.g.* “I don’t know, there’s just something about me, I wouldn’t use other people’s filters” [L04]). We defined the category ‘*preparedness*’ to include any purposive act which was carried out based on a clear understanding of foreseeable circumstances, and the express desire to mitigate or avoid risks associated with such circumstances (*e.g.* “There’s always been some kind of back-up, whether it’s been a bag of morphine pills or methadone or gear…I rarely, rarely let myself get sick” [I02]). Lastly, ‘*flexibility*’ was defined as resilience, or adaptability, particularly when practices were adapted or abandoned in the face of either imminent or future risk (*e.g.* “Even if I’m withdrawing really badly I would never share a work, I’d put it on a bit of foil [to smoke] – I would never, ever take someone’s works” [C04]). We outline below the protective practices falling within these three categories.

### Principles

#### Rules about not sharing injecting equipment, and/or the disposal of needles/syringes

Of the 37 participants 13 (35%) described having rules about not sharing injecting equipment or about disposing of needles/syringes. Among these 13 participants, rules included: never sharing filters (N = 4, 31%), not sharing needles/syringes (N = 4, 31%) or always using clean needles/syringes (N = 3, 23%), putting only one person’s sharps in the sharps container (N = 3, 23%), bending or snapping off the tip of the needle/syringe after use (N = 2, 15%), and not opening the sharps container to retrieve needles/syringes (N = 1, 8%).

#### Being in charge of mixing drugs and/or distributing the drug mix

Three (8%) of the 37 participants reported always being in charge of mixing drugs (*i.e.* the preparation of drugs to render them suitable to inject, generally by dissolving powdered heroin into citric acid or vitamin C), or the distribution of drugs among other PWID once they had been mixed.

#### Separation and/or marking of injecting equipment

Of the 37 participants in the study 17 (46%) described separating or marking their injecting equipment. Separating equipment included: meticulously allocating separate equipment for each user including a needle/syringe disposal bin in case it became necessary to re-use needles/syringes, hiding or locking up injecting equipment in the home, or injecting in different rooms in order to keep equipment separate. Marking included: burning needles/syringes, marking wrappers with initials, and habitually disposing of needles/syringes such that they could be identified if it became necessary to re-use (*e.g.* one partner habitually replaces the cap, while the other does not). The use of Nevershare™ syringes also indicated a desire to distinguish equipment as they are sold in multiple colours for the express purpose of reducing the risk of accidental sharing. [25] Separating equipment was practised by 10 (59%) of the 17 participants, four (24%) reported marking their equipment, and three (18%) both separated and marked their equipment.

#### Using drugs alone or at home

Of the 37 participants 23 (62%) reported a preference for using drugs alone or at home. Most participants described drug use alone or at home as a strong preference. Heroin was described by several participants as a drug best used alone, or as an anti-social experience, unlike using crack cocaine or marijuana which was described as a group, or a social activity.

### Preparedness

#### Stockpiling methadone/buprenorphine

Of the 37 participants, 11 (30%) described stockpiling methadone and/or buprenorphine (Subutex®). All 11 participants reported stockpiling methadone; two of these participants also reported stockpiling buprenorphine.

#### Carrying or stashing injecting equipment

Nearly half of the 37 participants (N = 17, 46%) reported always carrying injecting equipment and/or stashing needles/syringes. Of these 17 participants, 14 (82%) reported stockpiling clean needles/syringes, while nine participants (53%) reported always carrying injecting equipment.

### Flexibility

#### Temporary heroin smoking/snorting

Twenty-four (65%) of the 37 participants described having smoked/snorted heroin temporarily and as an alternative to injecting. For many participants smoking/snorting constituted their first experience using heroin; all went on to inject regularly, and the length of time participants smoked/snorted prior to injecting ranged from hours to 10 years. Several justifications for smoking heroin were often referenced by the same individual. Some participants smoked/snorted and injected concurrently.

### The Lived Context of Protection

#### Principles

Several key themes were identified from the narratives surrounding injecting principles. Factors which inspired routine and rigidly adhered to practices included: concerns about hygiene, personal responsibility, image management, injecting pragmatics, and risk management. Principles were often described as very personal commitments motivated by sensibilities developed in childhood; these included powerfully motivating concerns for hygiene, and notions of personal responsibility.

Concerns about hygiene were often described as transcendent, and did not apply specifically to drug use. Within the context of injection drug use, however, concerns for hygiene were primarily manifest as both an unwillingness to share syringes and/or other injecting equipment, and having strict rules about the disposal of syringes.

I’ve never knowingly shared works, but I have shared spoons and stuff and I know that’s an issue, but a lot of people I know that do share works, you know, wouldn’t dream of wearing somebody else’s fucking underwear or wearing somebody else’s socks, I’m just really squeamish about sharing works, I just find it horrible, the idea of it creeps me out. There’s something really personal about blood anyway and, apart from that, my mother was a nurse and I’ve got all my hang-ups about germs and stuff… [L16].

Notions of personal responsibility were often described in terms of responsibility to children or other close family members, though responsibility to other PWID was not uncommon.

Yeah, well there was rules, you want to come round, you make sure you clean up yourself and put it in the [sharps container]. I have a child…I have a nephew, he’s two years old, he’ll come walking around, you know what I mean? [C05].

Image management was another motivation for adherence to strict principles and was largely framed as a desire to avoid visible signs of drug use (particularly with respect to injection site infections), and to maintain a ‘normal’ looking image. This normalcy was described as important when it came to hiding drug use and avoiding negative attention and, often, stigmatisation at the hands of non-users (including hospital/clinic staff), and other PWID.

[I was smoking because] I didn’t like the marks all the time. It was difficult, like when I had relationships, like I’d show them the marks. I used to like going swimming and those marks are very hard to hide. So like I say, [I smoke] off and on, when I’ve been on my own I start using more, inject more than when I’m with a relationship or I have something going, so that’s what I’m saying. [I01].

Injecting pragmatics primarily involved concerns over vein care. Damaged or collapsed veins were a major concern as they resulted in lengthy and sometimes painful injecting experiences, and would often result in participants resorting to injecting veins that posed increased danger or discomfort (*e.g.* neck, groin).

Ah, I don’t really know anyone else who bends the tips [of used syringes] over. Why did I start doing that? I’ve been doing it for a long time…to stop myself using them again. To stop myself using them when they’re blunt really. To force myself to use a new one, you know. ’Cause it might look sharp, but I mean you know, it’s not sharp you know, and you’re scraping it to try and get it sharp and putting burs on it and you rip your veins up and everything like that, you know, so. It’s just, it’s just a safety thing, you know, to stop me using it and to stop anyone else using it as well. So no one else can use my, even my old ones. [I04].

Risk management was a common motivation for strict adherence to principles. The most common motivation was avoidance of HIV, though HCV and nondescript pathogens (*e.g.* “germs”, “diseases”, “the bug”) were also frequently mentioned.

Well yeah, the Hep-C is always a concern, but being heavily drugged a lot of the time things aren’t…things go out the window but, you know, the things you do you stick to, like clean [equipment], they’re just things you stick to. [L13].

Injection site infections were also a concern. Participants often described their injecting principles as having developed in response to family, friends, or injecting partners who had become ill – the physical, outward manifestations of illness were often explicitly referenced.

[My mother] had a big abscess and it even started going in to the, eating in to the bone, it was a big abscess…big abscess, and yeah, she had half her leg missing. My brother used to skin pop because he was diabetic and he was doing the gear as well, and he used to skin pop in his arm with his insulin and his gear, and he got an infection, I can’t remember, it was about 20 letters long…I’ve never shared, never, never shared a needle or a spoon in my life, if I share a needle, not a needle, a spoon, I’ll always make sure I draw it up first, know what I mean, and I make sure it’s all fresh stuff in there and I draw it up first, and then [someone else] can draw it second…I’ve never, never shared needles, no, never, or equipment, never, no, no. [C04].

No, because I never shared anything - or maybe I must have used J’s or one of the girls’ once - but other people’s, I don’t think I used other people’s, or if I did I washed it, because we used to wash, wash it out, because I never could use other people’s needles. And plus another thing, and plus I think it’s the yellow jaundice as well, because people used to go, because a couple of them used to go yellow, I says, ‘*woo, why?*’, right, and I think that’s what it really was because people used to get yellow jaundice from sharing needles and they used to get, some had blood poisoning and I think I just used to get frightened of getting disease germs. [L10].

Occasionally, participants were unable to describe their rigid adherence to certain principles, claiming that:

…it’s weird. I suppose, I was so used to [not sharing] that I just kept on doing it. And I suppose I wouldn’t have cared to have [to] share if I thought about it. But I was so used to doing it that I just…I kept on doing it. [L14].

#### Preparedness

Preparedness, in the form of stockpiling methadone/buprenorphine, or carrying or stashing injecting equipment, was commonly referred to as a principle or rule. Understanding contingencies, knowing ‘how it worked’, being organised, and anticipating need (particularly with respect to the need to inject upon waking in the morning) were all common themes among participants who reported having principles which involved preparedness. Anticipating need was occasionally described with considerable gravity; often with respect to the practice of stockpiling methadone/buprenorphine “in case of an emergency” [C01].

I didn’t want to catch any diseases…it just seemed to me logic that if syringes were free and they were in a clear label, which they were, there was no need really not to have them…you think ahead. That is just the way it worked for me. [I02].

I travelled a lot…but I always made sure I had clean syringes, or if I was going to be out on the road I made sure I had methadone if I knew that I like – you know what I’m saying, so, thank goodness I was never put in the position where I was really, really sick with heroin and no syringes. [I03].

Because he always had his, we always made sure we had enough of everything, we always made sure we had enough pins, enough citric, enough steri-wipes, enough of everything so that we never ran out, we used to have a little drawer full of pins and steri-wipes and citric and whenever we ran out we used to just go to the drawer and pick them up and fill our little bum bags back up again, it wasn’t…yeah, we’d always have equipment, always, always…we always used to get the equipment first, we always made sure we had equipment before we bought the drugs, that was, that’s a stupid thing to do is buy a drug if you haven’t got the equipment… if you’re going to take drugs organise man, make sure you’ve got your tools before you buy your drugs because it’s stupid…or smoke it if you haven’t got the tools, smoke it, simple, improvise, snort it. [L03].

#### Flexibility

Participants frequently described factors which disrupted protective practices. These factors were variously described as individual (*e.g.* a traumatic life event), situational (*e.g.* disruption of drug supply, exposure to risk-encouraging social networks, external threats to safety and/or security), and structural (*e.g.* barriers to accessing clean needles or foil, methadone distribution policy, obtrusive policing policy/practice). Often, despite the most assiduous planning and preparation, factors beyond the control of the participant frustrated protective practices. Despite this, many users were able to navigate such disruptions. The majority of the accounts of flexibility described finding other routes of administration when it was not deemed safe or desirable to inject. Participants reported smoking and/or snorting (and, in one case, taking heroin orally) when: in the company of people they did not know or trust, there was no access to clean/sharp syringes, or when injecting equipment other than syringes was being shared. The most common accounts detailed circumstances in which there was no access to clean/sharp needles and it became necessary to smoke or snort instead.

So I was waiting and waiting, I had the gear [in jail] and was waiting for hours and hours, and then I had to wait even ’till the next day because it was twenty-four hour [lock-up]. In the end they send it with the guy that was coming knocking on the door giving you a cup of tea in the evening. He had the [syringes] but [they were] used. And I’ve been waiting a day and a half already, and I say, ‘*just take it back’*. So I snorted it instead. [L14].

Additionally, participants claimed that being able to wait or abstain from using drugs was an effective strategy to avoid injecting in situations deemed risky.

If it was a choice between rummaging through a sharps bin that was just, you know, in a room that I’d never been in before in me life, and I show up in there with people I’d never met before in me life, I’d just take my gear and go…I’d fucking take my gear and go home. You know, I’ve walked like bloody eight miles, you know, eight miles just to score and [then have come] back… [I04].

Despite the fact that finding other routes of administration and being able to wait or abstain is likely prophylactic, the motivation for resorting to such measures was not always out of concern for contracting HIV or HCV. Participants frequently described smoking heroin to avoid: the dangers of injecting into the neck or groin, skin infections, and to avoid injuring veins.

But in the end you just end up saying, ‘*well look, you know, you look a mess’*, yeah you know, when you’re trying to get a vein in your hands and everything, mine are so much better now you wouldn’t believe, yeah. But I looked like a dart board honestly, I really did, it was horrible, you know, and um, I just started to smoke it, I just had to smoke more of it and that’s what I done. [L09].

## Discussion

We have used a qualitative life history approach to generate rich accounts of the protective strategies and associated motivations of PWID. This generated a descriptive typology of practices potentially protective against HCV contextualised by three distinct, but overlapping, strategic frameworks for action: *principles* framing normative practices in relation to minimising risk linked to injecting, an orientation towards *preparedness* and contingency planning to avoid disruption to risk management, and the capacity for *flexibility* to adapt when disruptions to normative practices or intentions occur. A life history approach enabled participants to narrate significant events in their lives, as well as to describe practices in relation to injecting and HCV, in time as well as in context. As a result, it becomes possible to establish roughly when motivations (and their associated practices) came into force, including how HCV-protective practices may have motivational antecedents unrelated directly to injecting (such as in the case of ideas around hygiene underpinning rules regarding not sharing). The life history methodology established that many practices were long-standing and remained consistent throughout the participant’s lifetime. Crucially, we find that many motivations surrounding practices which are potentially HCV-protective are indirectly rather than directly related to HCV, including situated concerns regarding hygiene, personal responsibility, image management, and maximising the pragmatics and pleasures of injecting.

Life history approaches have been used as a means to minimise recall bias in studies of patients suffering from chronic respiratory disease [26], and as a clinical tool to identify childhood maladaptations in patients undergoing psychodynamic psychotherapy. [27] Though life history approaches have been used in the field of addiction studies, it is often the case that life histories are elicited for the period after which dependent drug use has commenced. However, lengthy retrospective accounts commencing at the time of birth or early childhood have been used in addiction studies to identify and contextualise critical events in the lives of female crack users in Ohio [28], to identify the causal relationship between childhood experiences and heroin injection [29], and to highlight circumstances which might have been amenable to early intervention in deceased PWID in Scotland. [30] Here, we see the generation of life histories to prevent the narrowing of research focus specifically around risk as it relates to HCV, and to instead open up an understanding of how HCV risk, and its reduction, is lived in context. This gives rise to appreciating how motivations beyond the specifics of viral risk – such as preventing stigma by looking ‘normal’, or preventing painful injection by preserving veins through not using previously used needles, nonetheless have HCV-preventive potential. [21].

Relatively infrequent mention of HCV suggests that its avoidance may act as weak motivation for the multiple protective practices reported by participants. This could imply that HCV is not well understood in terms of behavioural prophylaxis, or that HCV avoidance is competing with other, often more immediate, motivations. The former possibility is contrary to the conclusion of Carruthers [31]; however, two studies among PWID in New York City reported that, though HIV avoidance was a primary motivator for engaging in strategies, practices and prevention tactics, relatively few participants (one out of 25) had extensive knowledge about HCV [32] and, among participants who did not have extensive knowledge, there were many misconceptions about transmissions, symptoms, long-term effects, and treatment. [33] The suggestion that HCV transmission is, in fact, well understood among PWID but that it is competing with more proximal concerns (*e.g.* risk of arrest, overdose, withdrawal) has been proposed by Harris *et al* [20], Harris and Rhodes [21], Rhodes and Treloar [22], and Roy *et al* [34]. Taken together, our findings suggest that HCV awareness and prevention interventions may have weak impact if juggled alongside multiple situated and competing priorities, and that the impact of HCV prevention efforts may be enhanced through social intervention approaches tailored in relation to the situated and pragmatic concerns of PWID.

### Study Limitations

The 95% confidence interval (25%–57%) for the HCV seropositive prevalence point estimate of 41% from the present study encompasses the prevalence estimate for both London (56%) [1], and England as a whole (45%). [1] However, as participation rates by HCV status could not be determined, the possibility that potential subjects who were seropositive for HCV would be less likely to participate in the study could not be established. Also, our assessment of the HCV status of the participants was limited to the sensitivity and specificity of the antibody and PCR tests used to determine HCV status.

Several of the participants who reported having consistently engaged in protective practices were confirmed as HCV positive, while others reporting a prolonged history of engaging in risky practices were confirmed as HCV negative. As study participants had lengthy injection careers an occasional, or atypical, change in injection behaviour could have resulted in HCV infection. These lapses may not have been reported as they may not have been conscious lapses, they may not have assumed much significance, or they may simply have been forgotten. Though the methodology used for this study was intended to minimise recall bias it is entirely possible that, owing to the sheer number of injection experiences in a participant’s injecting career, a few deviations may not have assumed much importance. It is also worth noting that all interview narratives are inevitably shaped by their social contexts, in which participants frame their accounts in light of perceived norms, including in relation to hygiene, personal responsibility, and safety. [35], [36] Furthermore, as participants were notified of the results of their serological screening following their initial interview, it is possible that confirmation of serostaus influenced participants’ perceptions of risk and may have thus influenced their responses during subsequent interviews. With respect to those who consistently engaged in risky practices, yet were found to be HCV negative, the possibility of cell-mediated immunity or of spontaneous seroreversion could not be ruled out. [37], [38].

Finally, as participants were recruited from HCV testing and screening services it is possible that the sample is not generalisable to the population, as PWID who avail themselves of these services may not share the risk profile of users who choose not to. Previous research among PWID in Australia [39], [40], Canada [41], and Europe [42] have suggested that the risk profile of PWID differs when comparing catchment facilities.

## Conclusion

In this sample of 37 PWID, protective practices had no obvious association with the outcome of interest (HCV status). Furthermore, avoiding HCV specifically was relatively infrequently cited as a motivation for engaging in protective practices. This highlights the need to consider how practices not directly related to viral avoidance may nonetheless have prevention potential, and the role of qualitative life history approaches in enabling these to become visible. Since these practices may also be effective in avoiding infection with HIV, or other blood-borne viruses, it is important to consider the relative importance of different motivations, with respect to specific practices, in order to formulate harm reduction interventions which appeal to the most pressing concerns of PWID.
